# Correction: Molecular networks and bioactivity-guided discovery of aliphatic derivatives from marine-derived fungus *Talaromyces* sp.

**DOI:** 10.1039/d6ra90140a

**Published:** 2026-10-01

**Authors:** Yaodong Ning, Yingxin Qu, Yingjie Zhou, Yao Xu, Xiaoyan Zhou, Binghua Jiao, Xiaoling Lu

**Affiliations:** a Department of Biochemistry and Molecular Biology, College of Basic Medical Sciences, Naval Medical University Shanghai 200433 People's Republic of China; b The First Affiliated Hospital of Naval Medical University Shanghai 200433 People's Republic of China

## Abstract

Correction for ‘Molecular networks and bioactivity-guided discovery of aliphatic derivatives from marine-derived fungus *Talaromyces* sp.’ by Yaodong Ning *et al.*, *RSC Adv.*, 2026, https://doi.org/10.1039/d6ra04846c.

The authors regret the inclusion of data in Table 2 for a compound that is not included in the article. The correct Table 2 is shown below.

**Table 1:** Antibacterial activities of compounds

Strain	1	2	3	4	Ampicillin
*B. subtilis*	64	>128	>128	>128	2
*S. aureus*	16	8	>128	32	2
*E. coli*	16	8	>128	16	2
*V. vulnificus*	>128	64	>128	>128	32
*V. anguillarum*	>128	>128	>128	>128	32
*V. parahaemolyticus*	>128	>128	>128	64	32

The authors would like to correct the following figure captions. The correct caption for Fig. 3 is “COSY and key HMBC correlations of compounds **1–3**.” The correct caption for Fig. 5 is “Calculated and experimental ECD spectra of compounds **1–3**.”

The authors would also like to correct the dry weights reported in Section 3.3. The correct sentence is “The dry weights of compounds **1–4** are 2.5 mg/2.1 mg/1.9 mg/3 mg, respectively.”

These changes were assessed and approved by an independent expert.

The Royal Society of Chemistry apologises for these errors and any consequent inconvenience to authors and readers.

